# Neurovascular coupling of striatal dopamine D_2/3_ receptor availability and perfusion using simultaneous PET/MR in humans

**DOI:** 10.1016/j.nsa.2024.104094

**Published:** 2024-10-05

**Authors:** Christian N. Schmitz, Xenia M. Hart, Moritz Spangemacher, Jana L. Roth, Ivana Lazarevic, Gunilla Oberthür, Karen A. Büsing, Robert Becker, Paul Cumming, Gerhard Gründer

**Affiliations:** a Department of Molecular Neuroimaging, Central Institute of Mental Health, Medical Faculty Mannheim, University of Heidelberg, Mannheim, Germany; b Department of Psychiatry and Psychotherapy, Central Institute of Mental Health, Medical Faculty Mannheim, University of Heidelberg, Mannheim, Germany; c Clinic for Radiology and Nuclear Medicine, University Hospital Mannheim, University of Heidelberg, Mannheim, Germany; d Department of Neuroimaging, Central Institute of Mental Health, Medical Faculty Mannheim, University of Heidelberg, Mannheim, Germany; e Center for Innovative Psychiatry and Psychotherapy Research (ZIPP), Central Institute of Mental Health, Mannheim, Germany; f German Center for Mental Health (DZPG), Partner Site Mannheim, Germany; g Department of Neuropsychiatry, Keio University School of Medicine, Tokyo, Japan; h Department of Nuclear Medicine, Bern University Hospital, Freiburgstraße 18, 3010, Bern, Switzerland; i School of Psychology and Counselling, Queensland University of Technology, Brisbane, QLD, 4059, Australia

**Keywords:** Dopamine, PET, Perfusion, pcASL, fMRI, Human

## Abstract

The midbrain dopamine system contributes to important neural functions in the basal ganglia, and is involved in aspects of pathological processes in schizophrenia. In preclinical and clinical studies, pharmacological blockade or stimulation of brain dopamine receptors alters cerebral perfusion, which is a surrogate marker of metabolic activity. However, there is scant documentation of this neurofunctional coupling in relation to individual differences in the dopamine system of healthy humans. We therefore tested the hypothesis that baseline dopamine D_2/3_ receptor availability predicts individual blood flow responses to challenge with a dopamine agonist.

We used [^18^F]fallypride positron emission tomography (PET) imaging to quantify dopamine D_2/3_ receptor availability as binding potential (BP_ND_) in nine healthy subjects. Using simultaneous perfusion-weighted functional magnetic resonance imaging (fMRI), we measured perfusion at baseline and after challenge with the dopamine agonist apomorphine.

Results of this multimodal imaging study revealed a strong negative association between baseline D_2/3_ dopamine receptor availability and apomorphine-induced perfusion changes in the human basal ganglia. There was considerable intra-individual variation in the neurovascular response to the apomorphine challenge, which may call for further investigation of the dopaminergic regulation of cerebral perfusion in patients with schizophrenia.

This study describes a novel paradigm for assessing dopamine sensitivity, facilitating an exploration of the dopamine supersensitivity hypothesis.

## 1 Introduction

The midbrain dopamine (DA) system is a major therapeutic target in diverse conditions such as schizophrenia, attention deficit hyperactivity disorder (ADHD), substance use disorders, and Parkinson's disease. There is broad clinical use of pharmaceuticals that modulate DA transmission, notably antipsychotic drugs (Efthimiou et al., 2024), psychostimulants (Caravaggio et al., 2021), and dopamine agonists (Dhall and Kreitzman, 2016; Scarselli et al., 2001). In recent decades, molecular imaging by positron emission tomography (PET), single photon computer tomography (SPECT) (Cumming, 2009), and functional magnetic resonance imaging (fMRI) (Bruinsma et al., 2018) have emerged as mature tools for investigating various aspects of cerebral DA in humans. The advent of multimodal PET/MRI imaging affords new prospects for probing relationships between neurotransmission and brain function changes (reviewed by (Sander et al., 2020)). However, there are relatively few multimodal imaging studies aiming to link DA markers with functional changes in brain activity (reviewed by (Calabro et al., 2023)).

Apomorphine is a non-selective DA receptor agonist with affinity for all DA receptor subtypes, which is known clinically for its transient efficacy in alleviating motor symptoms of Parkinson's disease (reviewed by (Jenner and Katzenschlager, 2016)). In a pre-PET era study, challenge with apomorphine increased regional cerebral blood flow (rCBF) in rat brain to the [^14^C]iodoantipyrine autoradiographic method (Ingvar et al., 1983). Nguyen et al. confirmed and extended this result in a pharmaco-fMRI rat study with apomorphine and amphetamine challenges (Nguyen et al., 2000). In a human single photon emission tomography (SPECT) study, apomorphine treatment increased rCBF in patients with Parkinson's disease, with the caveat that pretreatment with the putatively peripherally-acting antagonist domperidone blocked the effect (Sabatini et al., 1991). Conversely, administration of the DA D_2/3_ receptor (D_2/3_R) antagonist [^11^C]raclopride at a pharmacological dose evoking substantial receptor occupancy to PET increased cerebral blood volume to functional magnetic resonance imaging (fMRI) in nonhuman primate brain (Sander et al., 2013). In another preclinical fMRI study, direct optogenetic stimulation of DA neurons in the rat ventral tegmental area (VTA) increased the blood oxygenation level dependent (BOLD) signal of VTA-innervated limbic regions, including the ventral striatum (Lohani et al., 2017). Thus, a considerable body of evidence indicates that D_2/3_R signaling contributes to cerebrovascular regulation.

While BOLD contrast-based fMRI techniques tend to manifest baseline drift of the signal (Yan et al., 2009), arterial spin labelling (ASL) provides a more temporally stable reading of brain hemodynamic changes (Alsop et al., 2015). Thus, available results suggest that multimodal imaging of D_2/3_R availability to PET and cerebral perfusion to ASL-fMRI provide a valuable readout to evaluate the functional state in human brain.

Given this background, we used a hybrid PET/MR system to measure D_2/3_R availability to the antagonist ligand [^18^F]fallypride (FA), in conjunction with ASL measurements in a group of nine healthy participants. We thus applied pharmaco-PET/fMRI to test the hypothesis of dopamine sensitivity that baseline D_2/3_R availability in the striatum predicts baseline rCBF and individual apomorphine-induced changes in rCBF. As a secondary hypothesis, we tested for dopamine sensitivity also in the striatal subregions, as well as the thalamus and ITG. As such, we introduce a new methodology for investigating neurovascular coupling of the DA system in human brain using simultaneous PET/MR imaging.

## 2 Materials and methods

The local ethics committee of the Medical Faculty Mannheim of the University of Heidelberg (Mannheim, Germany) and the German Federal Office for Radiation Protection approved this investigation. We conducted the study in strict accordance with the ethical standards laid down in the 1964 Declaration of Helsinki in its most recent revision. All subjects gave written informed consent. All PET investigations were performed at the ZIPP core facility of the Central Institute of Mental Health Mannheim, Germany. This study has been registered at ClinicalTrials.gov as part of a larger study (ID: NCT03911726).

### 2.1 Subjects and study procedure

We initially recruited a group of 12 healthy subjects (six males and six females of mean ± SD age 33.8 ± 9.0 years (range 23–49 years). One participant experienced shivering and nausea as transient side effects associated with the pharmacological intervention, and had to be excluded from the study. We also excluded data of two other participants because of technical errors in the fMRI sessions. The analysis thus included nine healthy participants (four males and five females of mean ± SD age 34.6 ± 9.7 years (range 24–49 years). Detailed demographic data of the subjects are provided in Table 1 (and Supplementary Table 1 for all 12 participants).

**Table 1:** Demographic data of the included healthy subjects.

Parameter	Healthy Subjects (n = 9)
Age (years)	34.6 (±9.7)
Weight (kg)	74.2 (±14.7)
Education (years)	13.0 (±0.0)
Gender (m/f)	4/5
Handiness (right/left handed)	9/0
Smoker status (non-smoker/smoker)	9/0

All subjects were German-speakers with no evident speech pathology. Inclusion criteria consisted of non-pathological findings in standard laboratory parameters (electrolytes, liver parameters, creatine kinase, TSH, small blood count), electrocardiogram, as well as negative urine tests for substances of abuse (cannabis, amphetamines, cocaine, and opiates) on the days of screening and imaging. A pregnancy test at screening and just prior to imaging was mandatory for female subjects. All participants were carefully screened for presence of any psychiatric exclusion criterion using a structured diagnostic interview (**Mini** International Neuropsychiatric Interview (**M.I.N.I.**) for relevant DSM-5 axis I disorders (Sheehan et al., 1998). All subjects received compensation (300 Euro) for inconvenience arising from their participation.

All participants were under medical supervision during the PET recording and for at least additional 30 min thereafter.

### 2.2 Pharmacological challenge

Apomorphine is a partially selective DA receptor agonist, with higher affinity for D_2/3_Rs as compared to dopamine D_1_ receptors (Scarselli et al., 2001; Millan et al., 2002). Following earlier investigations of its effects on neurovascular coupling of DA D_2/3_Rs, we applied apomorphine (0.01 mg/kg body weight, s. c.), a dose affecting brain dopamine synthesis to PET, without pronounced side effects such as nausea (Jauhar et al., 2017).

### 2.3 Imaging

All patients underwent MRI on a high-resolution Siemens Biograph mMR (Siemens Healthineers, Germany), equipped with a 32 channel head coil. The MRI protocol included a structural T1 weighted image (MPRAGE) and perfusion weighted imaging sequences during the PET recording. T1-weighted structural images were obtained with a repetition time (TR) of 2 s, echo time (TE) = 2.58 ms, flip angle = 10°, 192 slices, slice thickness = 0.9 mm, voxel dimensions = 0.4 mm × 0.4 mm × 0.9 mm, FoV = 230 mm.

### 2.4 Radiochemistry and PET data acquisition

To measure D_2/3_R availability in the brain, we used the benzamide antagonist radioligand [^18^F]fallypride ([^18^F]FP), which was prepared in a high-yield modification of the radiosynthesis method for [^18^F]desmethoxyfallypride (Gründer et al., 2003; Vernaleken et al., 2013). Quality control of [^18^F]FP before administration entailed determination of chemical and radiochemical purity, molar activity, and pH.

**Fig. 1:**
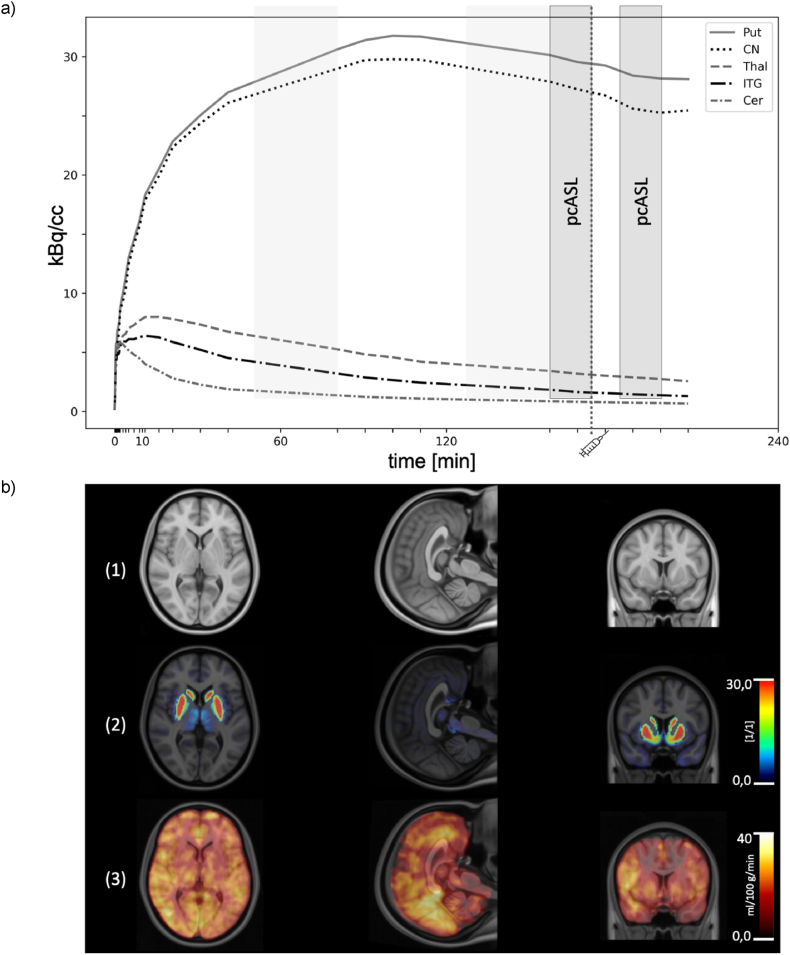
A) Experimental design: Representative time activity curves of the PET acquisition for the bilateral regions: putamen (Put), caudate nucleus (CN), thalamus, inferior temporal cortex (ITC) and cerebellum (Cer). The start of each time frame of the PET acquisition is denoted as tick on the horizontal axis. The time point of the administration of the dopamine agonist apomorphine is depicted as vertical dotted line. Periods of the perfusion weighted image acquisitions (ASL) are marked in dark grey. Inter-session pauses of PET-acquisition are marked in light grey, the signal of the inter-session pauses is based on linear interpolation. b) Representative parametric maps fused with a structural T1-weighted MR MNI152 NLIN 6th generation template (1), fusion parametric map of the dopamine receptor D_2/3_ binding potential combined with the structural T1-weighted MR MNI152 template (2) and fusion parametric map of cerebral blood flow combined with the structural T1-weighted MR MNI152 template (3).

PET data were acquired as dynamic emission images in 3D-mode (LR field-of-view 50 cm, AP FOV 26.55 cm). We injected a mean ± SD activity of 211 ± 14 MBq [^18^F]FP over 30 s into the cubital vein of the left arm. The corrected molar activity was 109 ± 34 (range 54–152) GBq/μmol, corresponding to < 1 nmol fallypride mass injected in each case. The injected activity did not significantly correlate with the BP_ND_ measured in any region of interest (ROI). Dynamic emission data were acquired during 210 min in a recording split into three consecutive blocks: 53.5 min of emission data, a 25-min break, 40 min of emission data, another 25-min break, and a final 60 min of emission data. Data was acquired as a series of up to 29-time frames (6 ∗ 10 s, 3 ∗ 20 s, 3 ∗ 1 min, 3 ∗ 2 min, 2 ∗ 5 min, and 13 ∗ 10 min, adapted from Slifstein et al. (2010) for the provision of an intermittent scanning-protocol; see Fig. 1, a). Data were reconstructed with an attenuation correction using an iterative high-resolution Flash 3D Vibe sequence with six iterations, based on a calculated MR-attenuation map (Martinez-et al., 2009). Each MR-AC map was resampled to PET resolution as part of the reconstruction, without additional filtering.

### 2.5 PET image processing

The dynamic PET-data was analyzed in the PMOD image analysis suite (PNEURO, PMOD Technologies LLC, Zürich, Schweiz, Version 4.4). Using a normalized mutual information algorithm as a dissimilarity function, we transformed each of the post-break segments of PET acquisition to match the T1-weighted MR-image of the first session. The concatenated dynamic PET image was denoised (Fast Non-Local Means (NLM)), motion corrected, and co-registered to the T1-weighted MR image of the first session. In cases of moderate head movement (interpolated session-breaks excluded), we performed frame-wise motion correction using a sequential registration procedure beginning with all frames spanning more than 1 min, while assuming no relevant head motion during the first 2 min of the recording. Upon using a Gaussian filter with a kernel of 6 mm and a squared difference sum as error function, we visually inspected the registration results by comparing outlines of the brain surface and of the striatal ROIs (caudate nucleus and putamen). The T1-weighted MR image was separately denoised with a fast Non-Local Means (NLM) algorithm, segmented into three probability maps (representing grey matter, white matter, and CSF), and normalized to the standard template (MNI152 NLIN 6th generation template).

We extracted time activity curves (TACs) from the preprocessed PET data using templates of polygonal ROIs applied to the spatially normalized dynamic recordings (Vernaleken et al., 2008, 2010, 2013). The bilateral brain regions of interest (ROIs) for putamen, caudate nucleus, thalamus, and inferior temporal cortex (ITC) were derived from the structural Harvard-Oxford atlas (Goldstein et al., 2007). A probabilistic cerebellar atlas served for extraction of the reference tissue TAC (Diedrichsen et al., 2011) (Fig. 1, a).

There was no discernible [^18^F]FP displacement after apomorphine treatment (Fig. 1, a) in accordance with Deutschländer et al. (2016), who saw no significant [^18^F]FP displacement in striatum of Parkinson's disease patients upon treatment with the DA agonist pramipexole. We calculated [^18^F]FP BP_ND_ of the respective ROIs using the simplified reference tissue model (SRTM), with cerebellum as the reference region (Lammertsma and Hume, 1996), using TACs terminating at 160 ± 4 min after tracer injection, i.e., just prior to the apomorphine administration. The cerebellum is validated and widely established as a reference region for [^18^F]FP BP_ND_ calculation by SRTM (Vernaleken et al., 2013; Cumming et al., 2013; Ishibashi et al., 2013), with the caveat that it contains a small D_2/3_R binding component (Hall et al., 1996). Based on prior studies, we expect a 5% bias in the estimation of striatal [^18^F]FA BP_ND_ arising from the cerebellum binding component (Gründer et al., 2003; Olsson et al., 2004). Following the method of Rousset et al., 1998, 2000, w e applied partial volume correction (PVC) of the selected brain TACs for the kinetic modelling.

### 2.6 Perfusion weighted imaging data acquisition

To evaluate the hemodynamic changes induced by the apomorphine challenge, we used a 5-post-labelling delay, pseudo-continuous arterial spin labeling (pcASL) protocol for perfusion-weighted imaging with background suppressed GRASE readout (Wang et al., 2013). Our protocol followed Alsop's white paper on the approach (Alsop et al., 2015); labeling pulse duration = 1.8 s, PLD = 0.5/1/1.5/2/2.5 s, TR = 2.5/3/3.5/4/4.5 s, FOV = 24 cm, matrix = 64 × 64, 40 × 3 mm slices, rate-2 GRAPPA factor = 2, TE = 45.46 ms, 8 pairs of tag and control for each delay, total scan time 11 min 53 s. The M0 calibration image was acquired with a TR of 4.4 s and otherwise analogous settings. The pcASL acquisition was performed twice per imaging session, i.e., immediately before and approximately 14 min after the apomorphine challenge. We selected these periods due to the pharmacokinetics of s. c. Apomorphine, which has a median time to maximum plasma concentration (T_max_) of 15–23 min (Agbo et al., 2021). We selected the multi-delay sequence based on its 15% lower error in rCBF estimation compared to a single‐PLD sequence (Woods et al., 2019). The labeling plane of the sequence was positioned with the recommended 90 mm distance from the center of imaging slices to the labeling plane perpendicular to the carotid and vertebral arteries. Due to the limited field of view of the sequence, parietal cortical areas were not included in the ASL acquisition. The analysis of the perfusion-weighted imaging was performed with the FSL toolbox Bayesian Inference for Arterial Spin Labeling MRI, BASIL (Chappell et al., 2009; Groves et al., 2009), using the PVC of Chappell et al. (2011), as required by the vulnerability of striatal fMRI signals to spillover. A similar analysis was performed for the average global grey and white matter voxel signals of brain perfusion, namely gCBFpre (before the pharmacological challenge) and gCBFpost (after the pharmacological challenge). These signals were compared using a paired *t*-test.

### 2.7 Perfusion image processing

Pharmacologically induced relative rCBF changes were determined by subtraction of the ROI-specific rCBF before and after the pharmacological challenge, with normalization to the baseline perfusion rCBFpre: ΔrCBF_ROI_ = (rCBFpost_ROI_ – rCBFpre_ROI_)/rCBFpre_ROI_.

### 2.8 Multimodal analysis

BP_ND_ and ΔrCBF values were extracted for each ROI. We used the Pearson correlation for comparison of regional BP_ND_ and ΔrCBF values, with significance level set at p = 0.05. The primary hypothesis focused on investigating BP_ND_ and ΔrCBF in the striatum. For the striatal subregions, as well as the thalamus and ITG, BP_ND_ and ΔrCBF were tested as secondary hypotheses. Multiple comparisons for the secondary hypotheses were corrected using the false discovery rate proposed by Benjamini and Hochberg (1995). We confirmed homoscedasticity and normality of the data with Levene's and Shapiro's tests, respectively. Median values of the ROI's BP_ND_ and ΔrCBF values served for the further analyses.

### 2.9 Physiological recording

To account for potential apomorphine-induced changes in heart rate and respiration rate, we used the patient monitoring unit (PMU) of the Siemens Biograph mMR (Siemens Healthineers, Germany). Based on custom-written code, we derived the average heart and respiration rates at baseline before the administration of the dopamine agonist and at 19 min after administration, which we compared by paired *t*-test.

### 2.10 Code availability

The dynamic PET-data were analyzed using the PMOD image analysis suite (PNEURO, PMOD Technologies LLC, Zürich, Schweiz, Version 4.4). In-house bash scripts for the analysis of the perfusion-weighted imaging based on the FSL toolbox Bayesian Inference for Arterial Spin Labeling MRI (BASIL), as well as Python codes for the statistical and correlation analysis and illustration of results are available upon request.

## 3 Results

The final study group consisted of nine participants (four males, five females) of mean ± SD age 35 ± 10 years, and 13 ± 3 years of education. All were right-handed and non-smokers.

### 3.1 [^18^F]FA BP_ND_ in healthy subjects

We summarize our experimental design, and present mean parametric maps and representative brain TACs in Fig. 1. There were no significant left-right asymmetries; mean ± SD BP_ND_ for the bilateral structures were 47.9 ± 4.0 in striatum (i.e., caudate-putamen), 50.5 ± 6.7 in caudate nucleus, 44.0 ± 4.5 in putamen, 3.2 ± 0.5 in thalamus, and 1.9 ± 0.5 in ITC (Table 2). There were a no significant correlations between striatal BP_ND_ and age (r = −0.3, p = 0.44), nor were there any associations of striatal BP_ND_ and sex (r = −0.4, p = 0.29) or the administered tracer dose (r = −0.2, p = 0.61).

**Table 2:** Dopamine D_2/3_ receptor availability (BP_ND_) with partial volume correction, regional baseline perfusion (rCBF) and apomorphine-induced perfusion changes (ΔrCBF) in the bilateral striatum, caudate nucleus (CN), putamen (Put), thalamus (Thal) and inferior temporal cortex (ITC) regions of interest (ROI) in (N = 9) healthy controls. P-values for the regions specified in the primary hypothesis are presented both with (p_FDR_) and without (p_uncorrected_) multiple comparison corrections, using the Benjamini and Hochberg method (Benjamini and Hochberg, 1995). Statistically significant values are marked with an asterisk (∗).

ROI	D_2/3_ binding potential		rCBF		ΔrCBF			
	BP_ND_	SD	$\frac{\text{mL}}{100\;g*\min}$	SD	Δ	SD	p_FDR_	p_uncorrected_
**Striatum**	46.9	4.0	61.1	24.3	8.08%	0.21		0.182
**CN**	50.5	6.7	58.4	20.2	9.28%	0.25	0.108	0.027
**Put**	42.4	4.8	79.9	31.5	6.87%	0.24	0.606	0.303
**Thal**	3.18	0.46	70.0	27.6	23.0%	0.21	0.627	0.470
**ITC**	1.87	0.45	54.7	21.1	−3.9%	0.25	0.743	0.743

### 3.2 Apomorphine-induced rCBF changes

There was no significant lateralization of baseline rCBF measures. At baseline, the average ± SD bilateral rCBF (mL/(100 g∗min)) was 61.1 ± 24.3 in entire striatum, 58.4 ± 20.2 in caudate nucleus, 79.9 ± 31.5 in putamen, 70.0 ± 27.6 in thalamus, and 54.7 ± 21.1 in ITC. After administration of apomorphine, there was no significant perfusion increase (ΔrCBF) in bilateral caudate-putamen (8.1 ± 21%; p_uncorrected_ = 0.18). To exploratory analysis, there was a 9.3 ± 25% increase in bilateral caudate nucleus that did not survive correction for multiple comparisons (p_uncorrected_ = 0.027; p_FDR_ = 0.11), and a non-significant 6.87 ± 24% increases in putamen (p_FDR_ = 0.61), a non-significant 3.0 ± 21% rCBF increase in bilateral thalamus (p_FDR_ = 0.63), and a non-significant 3.9 ± 25% decrease in bilateral ITC (p_FDR_ = 0.74) based on a paired *t*-test (Table 2).

### 3.3 Apomorphine-induced rCBF changes in caudate-putamen correlate with the [^18^F]FP BP_ND_

**Fig. 2:**
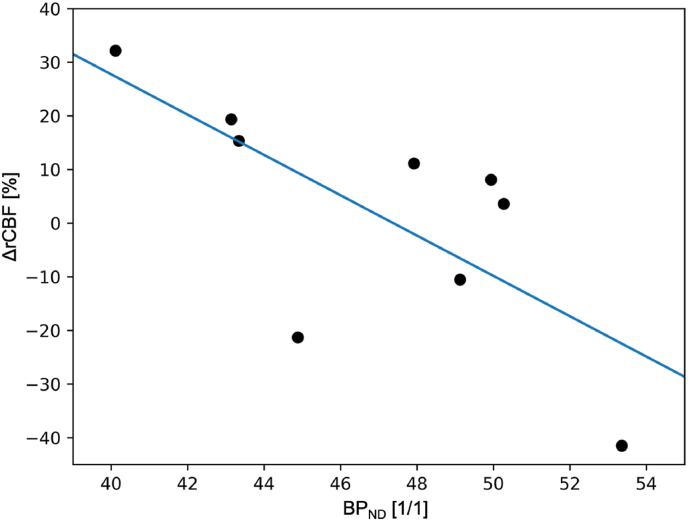
Change in cerebrovascular blood flow (ΔrCBF) in the bilateral striatum upon administration of the dopamine D_2/3_R agonist apomorphine as a function of the mean [^18^F]FP BP_ND_ in the group of nine healthy participants.

Pearson's correlation analysis showed significant negative correlations between [^18^F]FP BP_ND_ and ΔrCBF in the bilateral striatum (r = −0.71, p_uncorrected_ = 0.033), caudate nucleus (r = −0.64, p_FDR_ = 0.008), and thalamus (r = −0.66, p_FDR_ = 0.008). There were no significant correlations in putamen (r = −0.068, p_FDR_ = 0.79), or the ITC (r = −0.20, p_FDR_ = 0.79) (Fig. 2, Table 3).

**Table 3:** Correlation of dopamine D_2/3_ binding potential (BP_ND_) and apomorphine-induced perfusion changes of the bilateral regions of interest (ROI) striatum, caudate nucleus (CN), putamen (Put), thalamus (Thal) and inferior temporal cortex (ITC) in healthy controls (N = 9)). P-values for the regions specified in the primary hypothesis are presented both with (p_FDR_) and without (p_uncorrected_) multiple comparison corrections, using the Benjamini and Hochberg method (Benjamini and Hochberg, 1995). Statistically significant values are marked with an asterisk (∗).

ROI	*r*	p_FDR_	p_uncorrected_
**Striatum**	−0.71		0.033∗
**CN**	−0.64	0.008∗	0.004
**Put**	−0.07	0.787	0.787
**Thal**	−0.66	0.008∗	0.003
**ITC**	0.20	0.787	0.603

### 3.4 Apomorphine-induced cardiovascular and global gCBF effects

Previous studies have reported varying apomorphine-associated effects on heart rate and blood pressure (Rowbotham et al., 1983), or no changes of heart rate or blood pressure at all (Fagan et al., 2001; Stocchi et al., 2022). Comparison of mean heart and respiration rates at baseline and after apomorphine challenge (paired t-tests) showed no effect of treatment on heart rate (p = 0.50) or respiration rate (p = 0.46) (see Supplementary Fig. 1).

Comparison of global gCBF at baseline and after apomorphine challenge (paired t-tests) showed also no effect of treatment (p = 0.64).

## 4 Discussion

Our combined pharmaco-PET/fMRI study with apomorphine challenge in healthy subjects reveals neurovascular coupling of nigrostriatal dopamine system and local hemodynamic activity. In our study population of nine healthy participants, higher baseline D_2/3_ receptor availability predicted a lesser hemodynamic response in striatum.

### 4.1 [^18^F]FP BP_ND_ in healthy subjects

Our analysis of dynamic [^18^F]FP PET data during the first 196 min of the recording indicated a BP_ND_ of 46.9 ± 4.0 in bilateral striatum of healthy subjects, which exceeds our earlier estimates without PVC (e.g., 21.6 ± 4.3 (Cumming et al., 2013), 16.8 ± 3.0 (Rominger et al., 2012); for estimates of [^18^F]FP BP_ND_ without PVC, see Supplementary Table 2). Indeed, mouse dissection studies confirmed the substantial spillover of [^18^F]FP PET signal from striatum (Rominger et al., 2010). While PVC effects are naturally less pronounced at the scale of human striatum, present results with application of a PVC algorithm (Rousset et al., 1998, 2000) are in general accord with earlier reports of [^18^F]FP BP_ND_ in PV-corrected striatum (39.1 ± 7.0 (Kegeles et al., 2008); 33.0 ± 4.9 (Seaman et al., 2019)). Our PVC BP_ND_ findings in thalamus (3.18 ± 0.46) and ITC (1.87 ± 0.45) are likewise in agreement with the earlier PVC results in healthy humans (Kegeles et al., 2008; Seaman et al., 2019). While striatal dopamine D_2/3_R availability declines substantially with healthy aging (Wong et al., 1984), we did not see any significant decline with age in the present sample aged 24–49 years.

### 4.2 Global apomorphine-induced rCBF changes

In this study, the administration of the dopamine D_2/3_R-prefering full agonist apomorphine (mean dose 0.73 mg, i.e. 0.01 mg/kg) did not increase rCBF in the bilateral striatum, possibly due to high intra-individual variability of rCBF changes. In several earlier imaging studies (mainly in clinical populations) pharmacological challenges with dopamine agonists, and also antagonists, acutely stimulated rCBF in striatum and certain cortical regions (Chen et al., 2015; Fernández-Seara et al., 2011; Handley et al., 2013; Kobari et al., 1995; Mu et al., 2007; Schouw et al., 2013). In particular, Sabatini et al. found that apomorphine challenge (mean dose 0.3 mg) induced a widespread 12% increase in cerebral perfusion in Parkinson's disease patients (Sabatini et al., 1991). They proposed that extra-cerebrovascular dopamine receptors mediated this effect, based on their additional observation that pretreatment with the peripherally acting dopamine antagonist domperidone (60 mg, p. o.) blocked the perfusion increase. However, we note that the exclusively peripheral action of domperidone has been called into question (Ferrier, 2014). In a recent ASL study, challenge with the D_2/3_R partial agonist and 5HT_2A_ antagonist aripiprazole increased rCBF in striatum by 8% in first episode psychosis patients, with no such effects in their healthy control group (Bojesen et al., 2023). Such findings may represent sensitization of dopamine agonist responses in untreated patients with Parkinson's disease or untreated schizophrenia. Indeed, in a rat study using [^14^C]iodoantipyrine autoradiographic method, unilateral lesioning of the nigrostriatal dopamine pathway potentiated the apomorphine-induced rCBF increase (Ingvar et al., 1983).

Bojesen et al. reported no effect of the partial agonist on rCBF in their healthy control group (Bojesen et al., 2023). We report that the full dopamine D_2/3_R-preferring agonist apomorphine exhibits high intra-individual variability in rCBF among healthy human subjects, with a 9.3% increase in bilateral caudate nucleus activity. However, this increase did not survive correction for multiple comparisons (p_uncorrected_ = 0.027; p_FDR_ = 0.11). Given the small sample size as well as the high intra-individual variability in baseline and Δ10.13039/501100000928CBF values (see Fig. 2), we suppose that a larger sample size might support stronger claims about possible perfusion changes in other brain regions. Thus, further studies are required e.g. to validate extra-striatal effects.

### 4.3 Global apomorphine-induced rCBF changes correlate with the [^18^F]FA BP_ND_

Even though single subjects did not show systematic hemodynamic effects of the apomorphine on striatal rCBF, the single-subject trajectories could be predicted by the [^18^F]FA BP_ND_. To evaluate the association of [^18^F]FA BP_ND_ and the respective hemodynamic effect of apomorphine challenge, we calculated the correlation of [^18^F]FA BP_ND_ and apomorphine-induced rCBF changes in our group of healthy participants. There was a significant negative correlation (r = −0.71, p_uncorrected_<0.05) between baseline [^18^F]FA BP_ND_ in the bilateral striatum and the corresponding rCBF changes evoked by apomorphine challenge. Exploratory analysis revealed a negative correlation in thalamus (p_PVC_<0.003), but not in ITC. The negative coupling of D_2/3_R BP_ND_ and apomorphine-induced rCBF changes in striatal regions reported herein suggests that pharmacodynamic response to apomorphine depends on baseline D_2/3_R availability.

We confined our investigation to effects of dopamine agonism, whereas others have reported on perfusion effects of receptor antagonism. In a PET/MRI study in non-human primates, administration of non-tracer mass doses of the dopamine D_2/3_R antagonist [^11^C]raclopride increased striatal rCBF in ASL (Sander et al., 2013). Similarly, in human ASL studies, acute treatment with antipsychotic medications (i.e., D_2/3_R antagonists) increased rCBF. In particular, challenge with metoclopramide (10 mg, p. o.) increased rCBF in the striatum and thalamus in healthy volunteers (Fernández-Seara et al., 2011). In a recent study, haloperidol (3 mg, p. o.) and risperidone (0.5 and 2 mg, p. o.) both increased striatal rCBF in healthy volunteers as compared to placebo treatment (Selvaggi et al., 2019).

Thus, a compilation of present literature results with pharmacological challenge show that D_2/3_R agonists and antagonists both increased cerebral perfusion. This phenomenon implies a U-shaped relationship between rCBF and dopamine receptor activation. Such a relationship may have some bearing on the present finding that individuals with lowest striatal [^18^F]FA BP_ND_ showed the greatest changes in rCBF after apomorphine challenge (Fig. 2). We suppose that tonic dopamine signaling may be poised in healthy individuals such that there is a bimodal hemodynamic response to apomorphine challenge. Acting as an autoreceptor agonist, apomorphine (2–4 mg) inhibited the release of endogenous dopamine in the [^11^C]raclopride PET competition paradigm (de La Fuente-et al., 2001). Conceivably, the individuals with low striatal [^18^F]FP BP_ND_ in the present study may have high occupancy by endogenous dopamine, making them more sensitive to autoreceptor activation by the present apomorphine challenge. In this scenario, individual differences in autoreceptor tonus may have contributed to the variable hemodynamic responses in the present study group.

Although the pathway for hemodynamic responses to pharmacological challenges remains uncertain, present results in healthy individuals are pertinent to the concept of individualized medicine. Thus, responses in a given individual depend upon their particular state of dopaminergic signaling. Similarly, we have seen that neurochemical responses to challenge with antipsychotic medication depend upon baseline D_2/3_ receptor availability. Furthermore, prolonged treatment with antipsychotic medication can increase receptor binding (Silvestri et al., 2000), which might propagate to hemodynamic changes, perhaps as a marker for the emergence of receptor supersensitivity (Chouinard et al., 2017). Extension of present methods might illuminate the basis of dopaminergic supersensitivity as an emergent phenomenon upon repeated amphetamine administration in healthy controls and as a trait marker in patients with schizophrenia (Weidenauer et al., 2020).

### 4.4 Limitations

The small sample size of the group limits the power of detecting ROI-specific interaction effects, especially in extra-striatal regions of interest. Further exploration of the dose-dependence of hemodynamic responses to apomorphine in a larger population could validate our conjecture of a U-shaped relationship between hemodynamic response and the balance between dopamine D_2/3_ agonist and antagonist effects.

Due to the small sample size, the study has a higher risk of type II errors, where actual effects might go undetected. This limitation also makes the results less generalizable to broader populations. A larger sample could provide more robust evidence and allow for a more accurate analysis of dose-dependent effects, such as the hypothesized U-shaped relationship between hemodynamic responses to apomorphine and dopamine D_2/3_R BP_ND_.

## Author contributions

CNS, XMH and GG designed the research. XMH, JR, CS and MS recruited the participants. CS, XMH, MS, JR, KB and GO performed the research. GO and RB provided technical support and implemented the experimental setup. CS, JR and IL analyzed the data. CNS, MS, PC, and GG wrote the manuscript with input from all other authors. All authors gave final approval to the manuscript.

## Funding

Financial support for this work was provided by the department of Molecular Neuroimaging at the Central Institute of Mental Health Mannheim (CIMH).

## Competing interest

None to report.

## Declaration of competing interest

No potential competing interests to report.
