# Supplementary material for: Neurovascular coupling of striatal dopamine D2/3 receptor availability and perfusion using simultaneous PET/MR in humans
Source: Neurosci Appl. 2024 Oct 5;3:104094. doi: 10.1016/j.nsa.2024.104094 (PMC12244215; doi:10.1016/j.nsa.2024.104094)
Supplement: Multimedia component 1 [file mmc1.docx]

# SUPPLEMENTARY DATA

| **Parameter** | **Healthy Subjects (n=12)** |
| --- | --- |
| Age (years) | 33.8 (±9.0) |
| Weight (kg) | 73.7 (±20.6) |
| Education (years) | 12.8 (±0.4) |
| Gender (m/f) | 6/6 |
| Handiness (right/left handed) | 12/0 |
| Smoker status (non-smoker/smoker) | 12/0 |

**Supplementary table 1.** Demographic data of the screened healthy subjects (9 included, 2 excluded due to imaging malfunctions, 1 due to side effects of apomorphine challenge.

| **ROI** | **D_2/3_ receptor availability** | |
| --- | --- | --- |
|  | **BP_ND_** | SD |
|  |  |  |
| **CN** | 23.85 | 2.69 |
| **Put** | 25.92 | 2.72 |
|  |  |  |
| **Thal** | 2.38 | 0.33 |
| **ITC** | 1.20 | 0.31 |

**Supplementary table 2.** Dopamine D_2/3_ receptor availability (BP_ND_) without partial volume correction of the regions of interest (ROI) inferior temporal cortex (ITC), caudate nucleus (CN), putamen (Put) and Thalamus (Thal) in healthy controls (N = 9).


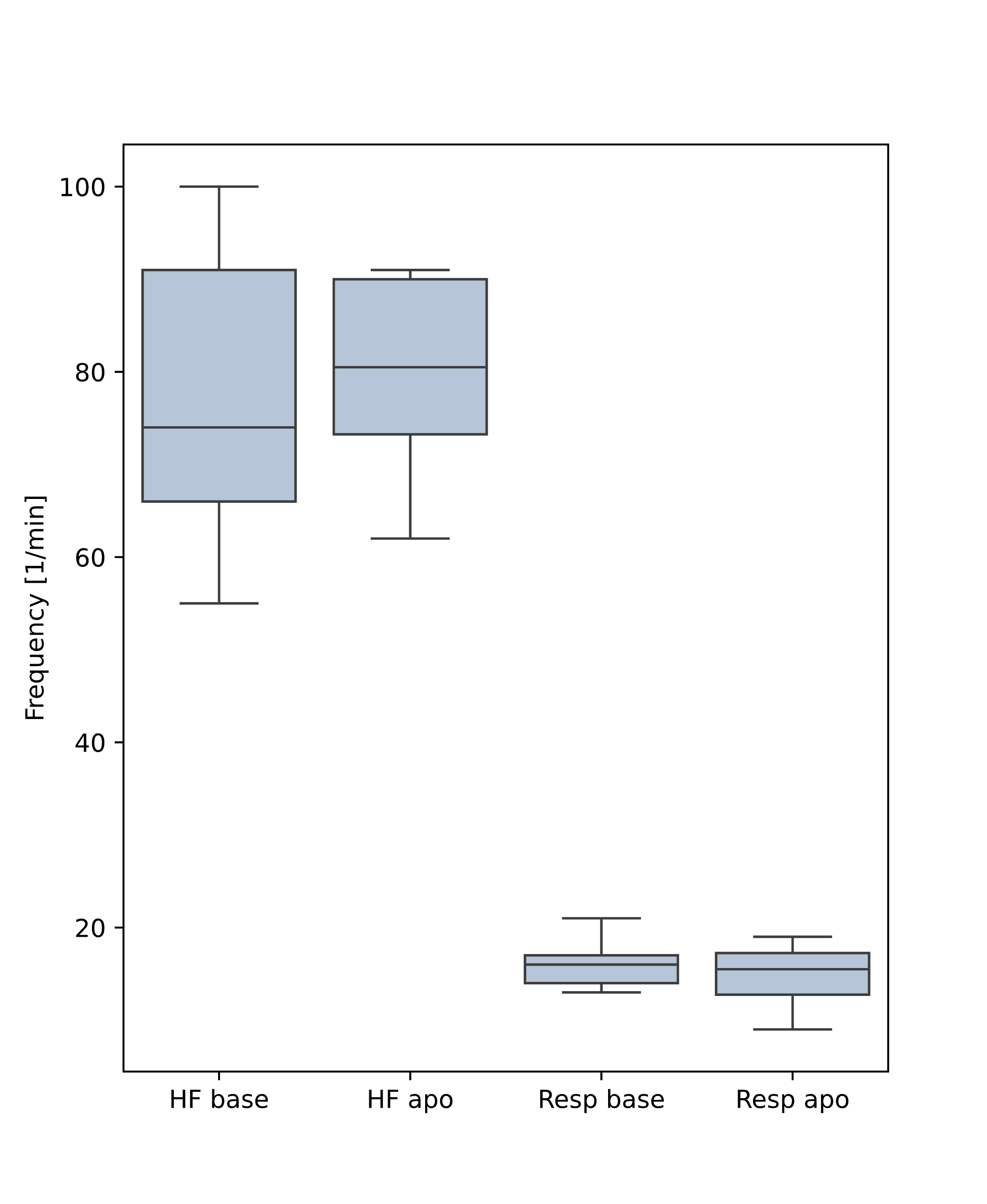


**Supplementary figure 1.** Average heart rate (HF) and respiration rate (Resp) at baseline (base), before the administration of the dopamine agonist and 19 minutes after the administration of apomorphine (apo) for all subjects.
